# Real-world survival benefit of adding durvalumab to chemotherapy in cholangiocarcinoma: a retrospective cohort analysis from a German Comprehensive Cancer Centre

**DOI:** 10.1016/j.esmorw.2026.100783

**Published:** 2026-09-21

**Authors:** S. Elbasan, S. Kasper, I.-A. Mavroeidi, K. Kostbade, J. Siveke, L. Spelsberg, S. Majorczyk, A. Neuhaus, S. Akbari, M. Wiesweg, H. Baba, S. Hartmann, H. Schmidt, U. Neumann, D. Albers, P. Markus, M. Schuler, I. Virchow

**Affiliations:** 1 Department of Medical Oncology, West German Cancer Centre (WTZ), University Hospital Essen, Essen, Germany; 2 Medical Faculty, University of Duisburg-Essen, Essen, Germany; 3 National Center for Tumor Diseases (NCT), NCT West, Essen, Germany; 4 Bridge Institute of Experimental Tumor Therapy (BIT), WTZ, University Hospital Essen, Essen, Germany; 5 Institute of Pathology, University Hospital Essen, Essen, Germany; 6 Department of Gastroenterology, Hepatology and Transplantation Medicine, University Hospital Essen, Essen, Germany; 7 Department of General, Visceral and Transplantation Surgery, University Hospital Essen, Essen, Germany; 8 Department of Internal Medicine and Gastroenterology, Elisabeth-Hospital Essen, Essen, Germany; 9 Department of General, Visceral and Trauma-Surgery, Elisabeth-Hospital Essen, Essen, Germany

**Keywords:** biliary tract cancer, cholangiocarcinoma, durvalumab, immune checkpoint inhibitor, overall survival, real-world data

## Abstract

**Background:**

Advanced biliary tract cancers (BTCs) carry a poor prognosis. Gemcitabine/platinum chemotherapy was the first-line standard until TOPAZ-1 and KEYNOTE-966 established immune checkpoint inhibitor-based combinations as a new standard. Real-world data with a chemotherapy-only comparator remain limited.

**Patients and methods:**

We retrospectively analyzed 172 patients with advanced BTC treated at the West German Cancer Centre (2018-2024): a durvalumab cohort (*n* = 64, treated 2022-2024) and a historical comparator cohort (*n* = 108, chemotherapy alone). Because cohort assignment was based on treatment era rather than randomization, propensity score matching (incorporating age, sex, metastatic burden, CA19-9, tumor location, and chemotherapy regimen) was carried out; analyses are reported for both the unmatched (*n* = 172) and matched (*n* = 77) cohorts. The primary endpoint was median overall survival (mOS); secondary endpoints included objective response rate (ORR), disease control rate (DCR), time to treatment failure (TTF), and toxicity.

**Results:**

In the matched cohort (*n* = 77), durvalumab was associated with significantly longer mOS (14.6 versus 11.1 months; hazard ratio 0.54, 95% confidence interval 0.307-0.956, *P* = 0.032) and a higher ORR (45% versus 20%; *P* = 0.015); DCR was numerically higher (65% versus 47.5%; *P* = 0.083). In the unmatched cohort (*n* = 172), durvalumab was associated with a larger mOS difference (18.7 versus 12.8 months; *P* = 0.003), higher ORR (42.2% versus 24.1%; *P* = 0.018) and DCR (65.6% versus 45.4%; *P* = 0.011); TTF trended in favor of durvalumab (4.8 versus 3.4 months; *P* = 0.066). No new safety signals were observed.

**Conclusions:**

These real-world data support the clinical benefit of adding durvalumab to gemcitabine/platinum chemotherapy in patients with advanced BTC, consistent with TOPAZ-1 and KEYNOTE-966. Given the retrospective design, findings should be regarded as supportive of, rather than exceeding, pivotal trial data.

## Highlights

• First real-world study of durvalumab in BTC with a chemotherapy-only comparator.
• Matched analysis: durvalumab improved OS versus chemotherapy (14.6 versus 11.1 months; HR 0.54).
• Propensity score matching was used to reduce confounding by treatment era.
• ORR was significantly higher with durvalumab in the matched cohort (45.0% versus 20.0%).
• No new safety signals; exploratory subgroup findings need prospective validation.

## Introduction

Cholangiocarcinoma (CCA) is a rare but highly lethal malignancy arising from biliary epithelial cells. It encompasses intrahepatic cholangiocarcinoma (iCCA), perihilar, and distal subtypes, as well as gallbladder cancer (GB), collectively termed biliary tract cancers (BTCs). Together, these account for ∼3% of all gastrointestinal malignancies yet carry a 5-year survival <10% in advanced stages.^1^ The majority of patients present with locally advanced or metastatic disease, precluding curative resection.

For more than a decade, the doublet of gemcitabine and cisplatin, established by the ABC-02 trial [median overall survival (mOS) 11.7 months], represented the backbone of first-line systemic therapy.^2^ Two landmark phase III trials have now transformed this paradigm. The TOPAZ-1 trial demonstrated that adding the programmed death-ligand 1 inhibitor durvalumab to gemcitabine/cisplatin significantly improved 24-month overall survival (OS) rate [24.9% versus 17.9%; hazard ratio (HR) 0.80, *P* = 0.021].^3^ Similarly, the KEYNOTE-966 trial showed a survival benefit for pembrolizumab added to gemcitabine/cisplatin (mOS 12.7 versus 10.9 months; HR 0.83, *P* = 0.0007).^4^ Based on these results, immune checkpoint inhibitor (ICI)−containing doublets became the new standard of care.

However, randomized trial data may not fully generalize to real-world populations with impaired performance status, comorbidities, or rare BTC subtypes, given strict trial eligibility criteria.^5^^,^^6^ Several real-world analyses have confirmed the feasibility of this regimen in unselected populations, including single- and multicenter series from Italy, Germany, and Austria^5^, ^6^, ^7^, ^8^, ^9^; however, all published real-world studies to date include only durvalumab-treated patients and lack comparison to chemotherapy-treated patients, limiting their ability to quantify the incremental benefit of adding immunotherapy in routine care.

We present a retrospective cohort analysis from the West German Cancer Centre (WTZ), University Hospital Essen—one of Germany’s largest oncological tertiary referral centers—comparing outcomes before and after the introduction of durvalumab in first-line BTC therapy.

## Materials and methods

### Study design and patient population

We conducted a retrospective single-center cohort study at the WTZ, University Hospital Essen. Patients were identified from the institutional Hospital Information System (KIS) by searching for the International Classification of Diseases-10 diagnosis codes for BTC (C22.1, C23, C24.0-C24.9) in combination with documented administration of systemic chemotherapy. Inclusion criteria were histologically confirmed advanced (unresectable, relapsed, or metastatic) BTC, with systemic therapy administered at the WTZ between May 2005 and January 2024. Patients had to be treated with first-line palliative chemotherapy with or without durvalumab. Two cohorts were formed for comparison: a historical cohort of patients treated with chemotherapy alone before the approval of durvalumab for BTC and a second cohort of patients treated with chemotherapy plus durvalumab following its approval.

### Data collection

Demographic, clinical, pathological, and treatment data were extracted from the electronic medical record of University Hospital Essen and transferred into a structured SPSS database. Data entries were cross-validated by a second researcher to ensure accuracy and completeness. Data were pseudonymized before analysis and all statistical analyses were carried out on the anonymized dataset. Variables included age, sex, primary tumor localization (iCCA, perihilar CCA, distal CCA/GB), CA19-9 level at start of palliative therapy, number of metastatic sites, first-line regimen (gemcitabine/platinum, gemcitabine monotherapy, other), response per RECIST v1.1^10^, date of progression, and date of death or last follow-up. Treatment response was evaluated retrospectively according to RECIST 1.1, based on the contrast-enhanced computed tomography or magnetic resonance imaging scans available in the institutional radiology archive. Response was assessed through comparison of baseline and follow-up imaging. Objective response rate (ORR) was defined as the percentage of patients with a complete or partial response as best response and disease control rate (DCR) was defined as the percentage of patients with a complete response, partial response, or stable disease at any follow-up assessment. Patients were eligible for response evaluation if at least one baseline and one follow-up radiological examination were available; patients without a qualifying follow-up scan were excluded from the response analyses.

### Treatment

All patients in the durvalumab cohort received durvalumab 1500 mg i.v. on day 1 of each 21-day cycle in combination with gemcitabine-based chemotherapy for up to eight cycles of chemotherapy followed by durvalumab maintenance. Dose reductions and delays were managed per institutional standard. Patients in the comparator cohort received first-line chemotherapy at the investigator’s discretion without immune checkpoint inhibitor therapy, comprising gemcitabine/platinum doublets, gemcitabine monotherapy, or other fluoropyrimidine-based regimens (FOLFOX and FOLFIRI) per institutional standards before the regulatory approval of durvalumab.

### Endpoints and statistical analysis

The prespecified primary endpoint was mOS (defined as time from first-line treatment initiation to death from any cause). Secondary endpoints were ORR (proportion achieving complete [CR] or partial response [PR]), DCR (proportion achieving CR, PR, or stable disease), and time to treatment failure (TTF), defined as the time from first-line treatment initiation to permanent discontinuation of therapy for any reason—including disease progression, unacceptable toxicity, death, or initiation of a subsequent line of therapy—whichever occurred first. Adverse events were retrospectively retrieved from electronic medical records and graded according to the Common Terminology Criteria for Adverse Events (CTCAE) v5.0. Particular attention was paid to immune-mediated adverse events (irAEs) attributable to durvalumab, including immune-mediated hepatitis, colitis, pneumonitis, endocrinopathies (thyroiditis, hypophysitis, and adrenal insufficiency), nephritis, dermatologic toxicity, and other rare immune-related events. irAEs were further categorized by severity (any grade, grade ≥3). Survival analyses used the Kaplan−Meier method; between-group comparisons used the log-rank test. Explorative subgroup analyses of OS and TTF were carried out across four dimensions: sex, age group, tumor localization, and disease status at treatment initiation using univariate Cox proportional hazards regression models, with HRs and 95% confidence intervals (CIs) calculated separately for each subgroup stratum. Response differences were evaluated with Fisher’s exact test. A two-sided *P* < 0.05 was considered statistically significant. All analyses were carried out with SPSS v29.0 (IBM, Armonk, NY).

### Propensity score matching

To reduce the effect of confounding arising from the nonrandomized, time-period–based cohort assignment, propensity scores were estimated using multivariable logistic regression with treatment group (durvalumab versus comparator) as the dependent variable. Covariates included age (continuous), sex (male versus female), number of metastatic sites (0-1 versus >1), CA19-9 level at the start of palliative therapy (log-transformed to account for right-skewed distribution), primary tumor location [intrahepatic cholangiocarcinoma versus other (perihilar/distal CCA, GB)], and first-line chemotherapy regimen (gemcitabine/platinum-doublet versus other). Patients were matched 1 : 1 without replacement using nearest-neighbor matching on the logit of the propensity score. All subsequent comparative analyses were carried out on the matched cohort.

### Ethics approval

The study protocol was approved by the Ethics Committee of the Medical Faculty of the University of Duisburg-Essen (approval no. 15-6497-BO). As this was a retrospective, noninterventional study of pseudonymized, routinely collected clinical data, the requirement for written informed consent was waived in accordance with § 15 of the professional code of conduct for physicians in North Rhine-Westphalia (Berufsordnung für die nordrheinischen Ärztinnen und Ärzte, MBO-Ä) and § 6 (2) of the North Rhine-Westphalian Health Data Protection Act (Gesundheitsdatenschutzgesetz Nordrhein-Westfalen, GDSG NRW). All patient data were extracted from the electronic KIS of UH Essen, pseudonymized before analysis, and stored in accordance with institutional data governance regulations.

## Results

### Patient characteristic

A total of 212 assessable patients were identified from the KIS of the University Hospital Essen. Of these, 64 patients had received treatment during the durvalumab-approval period and 148 patients had received treatment during the preceding, non-durvalumab period. Within the non-durvalumab period, 40 patients were excluded because chemotherapy had been administered with adjuvant rather than palliative intent, leaving 108 patients eligible as the comparator cohort (Supplementary Figure S1, available at https://doi.org/10.1016/j.esmorw.2026.100783). Two nonoverlapping cohorts were thus defined by treatment era: the durvalumab cohort (*n* = 64) received gemcitabine-based therapy plus durvalumab from July 2022 onward; the comparator cohort (*n* = 108) received chemotherapy alone in the period preceding regulatory approval (Figure 1). Baseline characteristics are summarized in Table 1. Median age was 65.5 years (range, 26.9-84.7) in the durvalumab group and 60.7 years (range, 29.2-81.3) in the comparator cohort. The cohorts showed marginal numerical imbalances: female patients predominated in the durvalumab group (57.8%), whereas males predominated in the comparator group (59.3%). The most common tumor subtype was iCCA, accounting for 75% of the durvalumab cohort and 57.4% of the comparator cohort. Median CA19-9 at treatment initiation was markedly higher in the durvalumab cohort than in the comparator cohort [434 U/ml (range, 1.2-230 486) versus 85 U/ml (range, 1-356 755); *P* = 0.029]. The proportion of patients with CA19-9 above the upper limit of normal was also numerically higher in the durvalumab group (73.4% versus 39.5%; *P* = 0.102); however, CA19-9 data were missing in a substantially larger proportion of the comparator cohort (35.1%) than of the durvalumab cohort (1.6%). The distribution of metastatic sites (0-1 versus >1) was comparable between groups (51.6% versus 50.9% with 0-1 metastatic sites; *P* = 0.355), with missing data in 8.8% of the comparator cohort and none in the durvalumab cohort. Gemcitabine/platinum-based chemotherapy was administered to all durvalumab-treated patients and to 62% of the comparator cohort; 21.3% of the comparator cohort received gemcitabine monotherapy and 16.7% received other regimens (FOLFOX, FOLFIRI, or trial-based combinations).

**Figure 1:**
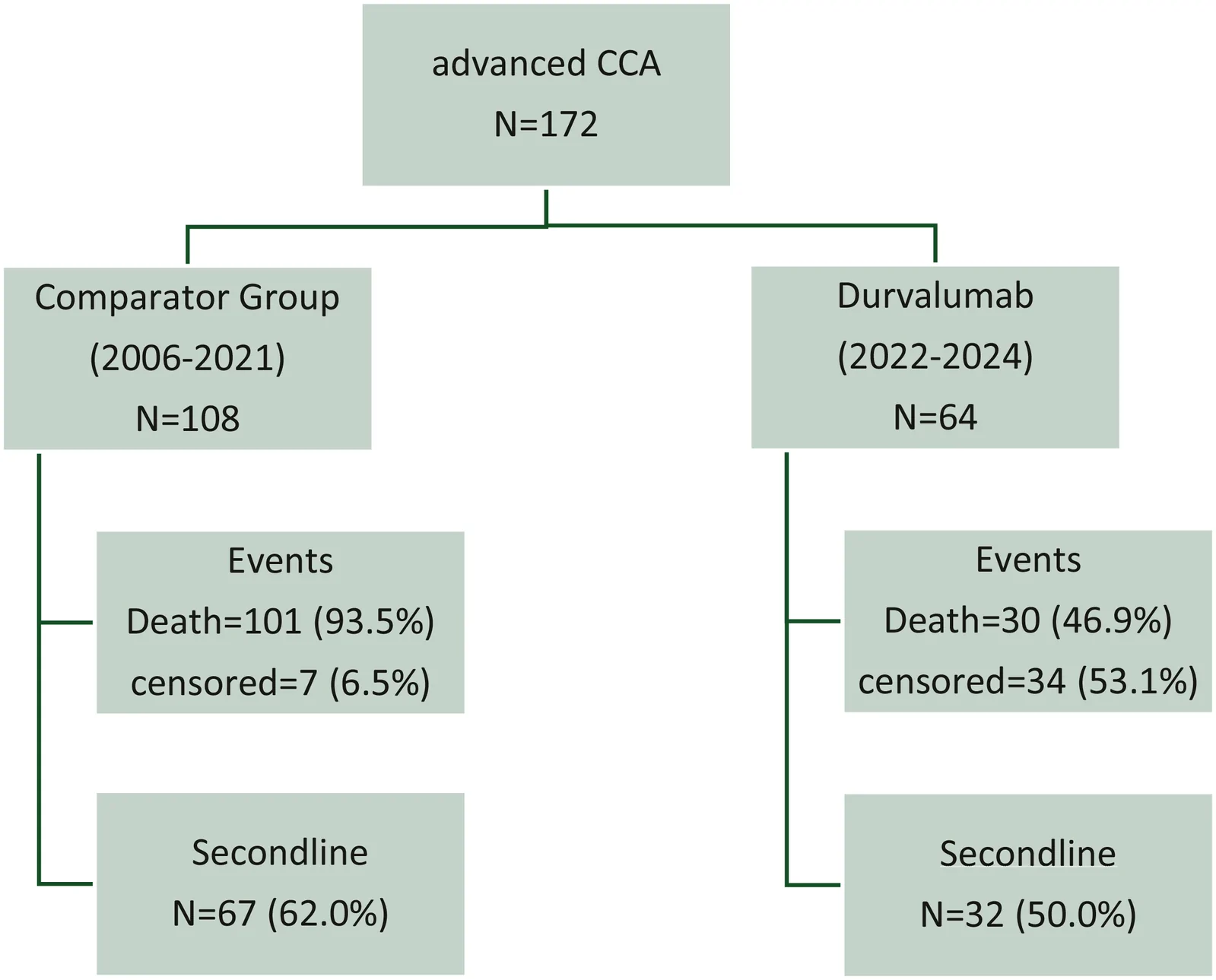
**CONSORT diagram of the study population.** From a total of 172 patients with advanced cholangiocarcinoma (CCA) treated with first-line systemic therapy at the WTZ between 2006 and 2024, 108 patients received chemotherapy alone (comparator cohort, 2006-2021) and 64 patients received gemcitabine/platinum plus durvalumab (durvalumab cohort, 2022-2024). Death events occurred in 101/108 (93.5%) comparator patients and 30/64 (46.9%) durvalumab patients; 7 (6.5%) and 34 (53.1%) were censored, respectively. Second-line therapy was administered to 67/108 (62%) and 32/64 (50%) of patients, respectively.

**Table 1:** Baseline patient characteristics

Characteristic	All patients (*N* = 172)	Durvalumab (*n* = 64)	Comparator cohort (*n* = 108)	*P*
Median age, years (range)	62.6 (26.9-84.7)	65.5 (26.9-84.7)	60.7 (29.2-81.3)	0.093^a^
Sex				0.040^b^
Female	82 (47.7%)	37 (57.8%)	44 (40.7%)	
Male	90 (52.3%)	27 (42.2%)	64 (59.3%)	
Tumor subtype				
iCCA	110 (64%)	48 (75%)	62 (57.4%)	0.022^b^
Perihilar CCA	26 (15.1%)	5 (7.7%)	21 (19.4%)	0.048^b^
Distal CCA/GB	32 (18.6%)	11 (17.2%)	21 (19.4%)	0.840^b^
n.d.	4 (2.3%)	0 (0%)	4 (3.7%)	0.298^b^
Metastatic sites				
0-1	91 (51.1%)	33 (51.6%)	58 (50.9%)	0.355^b^
>1	77 (43.3%)	31 (48.4%)	46 (40.4%)	
n.d.	10 (5.6%)	0 (0%)	10 (8.8)	
CA19-9 level				
Median U/ml (range)	178 (1-356 755)	434 (1.2-230 486)	85 (1-356 755)	0.029^a^
>ULN	92 (51.7%)	47 (73.4%)	45 (39.5%)	0.102^b^
n.d.	41 (23%)	1 (1.6%)	40 (35.1%)	
First-line regimen				
Gemcitabine/platinum ± durvalumab	131 (76.2%)	64 (100%)	67 (62%)	<0.001^b^
Gemcitabine monotherapy	23 (13.4%)	0 (0%)	23 (21.3%)	<0.001^b^
Other (FOLFOX, FOLFIRI, trial)	18 (10.5%)	0 (0%)	18 (16.7%)	<0.001^b^

iCCA, intrahepatic cholangiocarcinoma; GB, gallbladder cancer; n.d., not determined.

a Mann–Whitney *U*-test.

b Fisher’s exact test.

### OS

Patients treated with durvalumab-based chemoimmunotherapy had a significantly longer mOS of 18.7 months (95% CI 13.1-24.4) as compared with patients treated with chemotherapy alone (mOS 12.8 months, 95% CI 9.2-16.4, log-rank *P* = 0.003; Table 2 and Figure 2A), corresponding to an absolute median survival gain of 5.9 months (HR 0.533, 95% CI 0.351-0.810, *P* = 0.003). Subgroup analyses by anatomical localization revealed a particularly pronounced benefit in patients with iCCA, in whom mOS nearly doubled with durvalumab (19.8 versus 11.1 months; HR 0.475, 95% CI 0.284-0.795, *P* = 0.003), whereas non-iCCA tumors (perihilar, distal, or GB) showed comparable outcomes (14.3 versus 14.0 months; HR 0.783, 95% CI 0.374-1.639, *P* = 0.507), suggesting the benefit is largely driven by the iCCA subgroup (Table 2, Figure 2B and C and Supplementary Figure S2, available at https://doi.org/10.1016/j.esmorw.2026.100783). The OS benefit was not explained by differences in postprogression (second-line) therapy rates, which were comparable between cohorts.

**Table 2:** Efficacy outcomes: survival and response rates

Outcome	Durvalumab (*n* = 64)	Comparator cohort (*n* = 108)	*P*
mOS, months (95% CI)	18.7 (13.1-24.4)	12.8 (9.2-16.4)	0.003
HR OS (95% CI)	0.533 (0.351-0.810)		0.003
iCCA subgroup	19.8 (7.1-32.5)	11.1 (5.7-16.5)	0.202
Non-iCCA subgroup	14.3 (7.0-21.5)	14.0 (11.1-17.0)	0.850
Median TTF, months (95% CI)	4.8 (3.6-5.9)	3.4 (2.1-4.7)	0.066
HR TTF (95% CI)	0.727 (0.516-1.025)		0.069
ORR (CR + PR)	42.2%	24.1%	0.018
DCR (CR + PR + SD)	65.6%	45.4%	0.011

CI, confidence interval; CR, complete response; DCR, disease control rate; HR, hazard ratio; iCCA, intrahepatic cholangiocarcinoma; mOS, median overall survival; ORR, objective response rate; OS, overall survival; PR, partial response; TTF, time to treatment failure.

**Figure 2:**
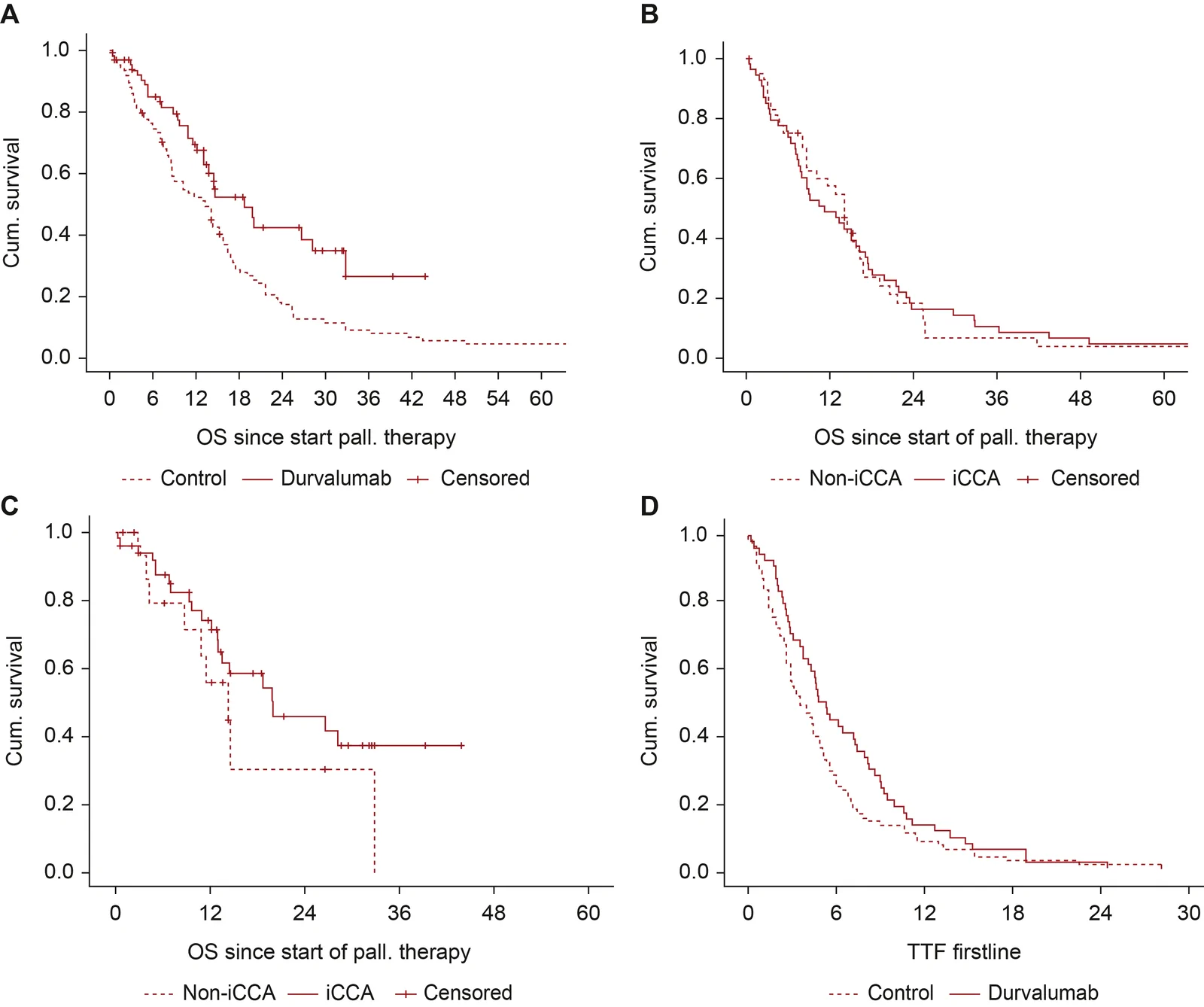
**(A) Kaplan–Meier curves for OS in the overall study population.** Comparison between patients receiving gemcitabine/platinum plus durvalumab (durvalumab cohort; *n* = 64) and those receiving chemotherapy alone (comparator cohort; *n* = 108). mOS: 18.7 months (95% CI 13.1-24.4) versus 12.8 months (95% CI 9.2-16.4); log-rank *P* = 0.003. Tick marks indicate censored observations. (B) Kaplan–Meier curves for OS by tumor subtype within the comparator cohort (*n* = 108). Comparison between intrahepatic CCA (iCCA; solid line) and non-iCCA (perihilar, distal, gallbladder; dashed line). mOS: iCCA 11.1 months (95% CI 5.7-16.5) versus non-iCCA 14.0 months (95% CI 11.1-17.0); log-rank *P* = 0.850. (C) Kaplan–Meier curves for OS by tumor subtype within the durvalumab cohort (*n* = 64). Comparison between iCCA (solid line) and non-iCCA (dashed line). mOS: iCCA 19.8 months (95% CI 7.1-32.5) versus non-iCCA 14.3 months (95% CI 7.0-21.5); log-rank *P* = 0.202. The most pronounced absolute survival gain with durvalumab was observed in the iCCA subgroup (19.8 versus 11.1 months when compared with iCCA controls). (D) Kaplan–Meier curves for TTF. Comparison between the durvalumab cohort (*n* = 64) and comparator cohort (*n* = 108). Median TTF 4.8 months (95% CI 3.6-5.9) versus 3.4 months (95% CI 2.1-4.7); log-rank *P* = 0.066. CI, confidence interval; iCCA, intrahepatic cholangiocarcinoma; mOS, median overall survival; OS, overall survival; TTF, time to treatment failure.

### TTF

Median TTF, capturing therapy discontinuation for any reason, was 4.8 months (95% CI 3.6-5.9) with chemoimmunotherapy as compared with 3.4 months (95% CI 2.1-4.7) in the chemotherapy cohort (log-rank *P* = 0.066; Table 2 and Figure 2D). This corresponds to a HR of 0.727 (95% CI 0.516-1.025, *P* = 0.069).

### Subgroup analyses

Subgroup analyses of OS and TTF showed a consistent trend favoring durvalumab across strata, reaching statistical significance for OS in male sex (HR 0.41, 95% CI 0.21-0.78, *P* = 0.006), age ≥65 years (HR 0.49, 95% CI 0.26-0.92, *P* = 0.026), iCCA (HR 0.46, 95% CI 0.28-0.80, *P* = 0.005), and unresectable/metastatic disease (HR 0.55, 95% CI 0.35-0.86, *P* = 0.009) (Figure 3 and Supplementary Figures S3 and S4, available at https://doi.org/10.1016/j.esmorw.2026.100783). For TTF, HRs trended numerically in favor of durvalumab across subgroups, reaching significance only in recurrent disease (HR 0.25, 95% CI 0.07-0.92, *P* = 0.036), with subgroup HRs generally closer to 1.0 than for OS.

**Figure 3:**
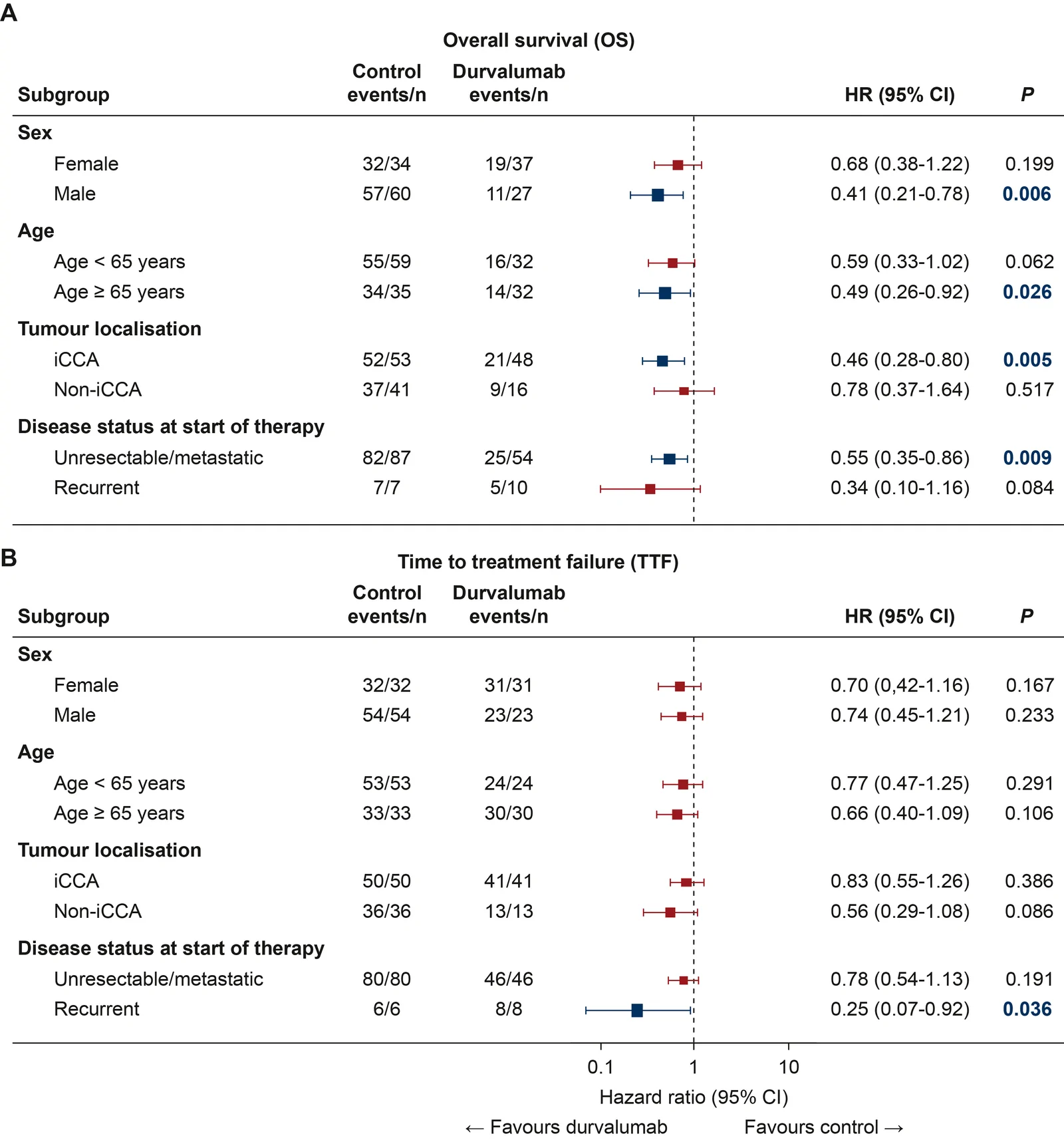
**Forest plots of subgroup analyses for OS (A) and TTF (B) in the durvalumab cohort versus the comparator cohort.** HRs with 95% CIs are shown for prespecified subgroups: sex, age (<65 versus ≥65 years), tumor localization (iCCA versus non-iCCA), and disease status at start of therapy (unresectable/metastatic versus recurrent). Dark blue squares indicate statistically significant subgroups (*P* < 0.05); gray squares indicate nonsignificant subgroups. Square size is proportional to the number of events. Dashed vertical reference line at HR = 1.0. HR < 1 favors durvalumab. CI, confidence interval; HR, hazard ratio; iCCA, intrahepatic cholangiocarcinoma; OS, overall survival; TTF, time to treatment failure.

### Response rates

ORR according to RECIST 1.1 was significantly higher in the durvalumab-treated cohort at 42.2% as compared with 24.1% in patients receiving first-line chemotherapy (Fisher’s exact *P* = 0.018) (Table 2). Similarly, DCR reached 65.6% with durvalumab versus 45.4% with chemotherapy alone (*P* = 0.011).

### Safety and immune-mediated toxicity

Given the retrospective nature of this analysis, adverse events were not systematically documented across all patients and all grades. Accordingly, the safety analysis focused on clinically relevant toxicities of CTCAE grade ≥3, as these are the events most consistently and reliably captured in routine medical records. Also, they carry the greatest impact on treatment decisions and patient outcomes. Grade 1-2 events are likely substantially under-reported in this setting and are therefore not presented. Safety data for grade ≥3 events are summarized for both cohorts in Table 3, with irAEs reported separately. The overall frequency of severe adverse events was broadly comparable between durvalumab-treated patients and with the comparator cohort. No unexpected toxicity patterns were identified. Hematotoxicity of grade ≥3 was the most common severe adverse event in both groups [durvalumab: 13 patients (20.3%); comparator cohort: 16 patients (14.8%)], encompassing neutropenia, anemia, and thrombocytopenia. Grade ≥3 hepatotoxicity was observed in 11 patients (17.2%) in the durvalumab cohort and 16 patients (14.8%) in the comparator cohort. Grade ≥3 nephrotoxicity was rare and observed in one durvalumab-treated patient (1.6%) only. Infections with concurrent neutropenia were infrequent in both cohorts [comparator group: 3 (2.8%); durvalumab: 2 (3.1%)]. Other severe nonhematological toxicities included neuropathy, diarrhea, and nausea in ≤2 patients each per cohort. Regarding irAEs, these are by definition restricted to the durvalumab cohort. A total of 10 patients (15.6%) experienced irAEs of any grade. The spectrum of irAEs was consistent with the known durvalumab safety profile. The majority were of low grade (CTCAE grades 1-2) and managed with dose interruption or systemic corticosteroids per institutional protocols. No treatment-related deaths were observed.

**Table 3:** Summary of safety data

Parameter	Durvalumab (*n* = 64)	Comparator cohort (*n* = 108)
Adverse events—*n* (%)		
Hematotoxicity ≥ grade 3	13 (20.3)	16 (14.8)
Hepatotoxicity ≥ grade 3	11 (17.2)	16 (14.8)
Nephrotoxicity ≥ grade 3	1 (1.6)	0 (0)
Infections with neutropenia	2 (3.1)	3 (2.8)
Neuropathy ≥ grade 3	1 (1.6)	1 (0.9)
Diarrhea ≥ grade 3	0	1 (0.9)
Nausea ≥ grade 3	1 (1.6)	1 (0.9)
irAEs		
Any grade	10 (15.6)	

irAEs, immune-related adverse events.

### Propensity score matching

To account for the nonrandomized, time-period–based assignment of patients to treatment groups, propensity scores were estimated for the durvalumab and the comparator cohort using a multivariable logistic regression model incorporating age, sex, number of metastatic sites, CA19-9 level, primary tumor location (iCCA versus rest), and first-line chemotherapy regimen (gemcitabine/platin doublet versus rest) as covariates. Patients were matched 1 : 1 without replacement using nearest-neighbor matching on the logit of the propensity score. This yielded a matched cohort of 77 patients (durvalumab, *n* = 40; comparator, *n* = 37). In the matched cohort, patients treated with durvalumab plus chemotherapy had a significantly longer mOS than those treated with chemotherapy alone [14.6 months (95% CI 7.7-21.4) versus 11.1 months (95% CI 5.0-17.3), HR 0.54 (95% CI 0.307-0.956), log-rank *P* = 0.032; Supplementary Table S1 and Figure S6A, available at https://doi.org/10.1016/j.esmorw.2026.100783]. Median TTF was numerically longer in the durvalumab group [4.7 months (95% CI 3.6-5.9) versus 3.4 months (95% CI 2.1-4.7)], although the difference did not reach statistical significance [HR 0.739 (95% CI 0.463-1.178), log-rank *P* = 0.2; Supplementary Table S1 and Figure S6B, available at https://doi.org/10.1016/j.esmorw.2026.100783]. The ORR was significantly higher with durvalumab (45% versus 20%; *P* = 0.015) and the DCR was numerically higher (65% versus 47.5%; *P* = 0.083; Supplementary Table S1, available at https://doi.org/10.1016/j.esmorw.2026.100783).

### Cross-trial comparison

Efficacy outcomes were benchmarked against the pivotal phase III trials TOPAZ-1 and KEYNOTE-966 (Supplementary Table S2, available at https://doi.org/10.1016/j.esmorw.2026.100783). mOS in our durvalumab cohort (18.7 months) numerically exceeded both trials (12.8 and 12.7 months, respectively), while mOS in our comparator cohort (12.8 months) closely mirrored their placebo arms (11.5 and 10.9 months), supporting the representativeness of our patient population. ORR with durvalumab (42.2%) likewise exceeded both trials (26.7% and 29%), whereas DCR was lower (65.6% versus 85.3% and 75%); these comparisons are interpreted in the Discussion.

## Discussion

This retrospective analysis from a large German Comprehensive Cancer Centre describes outcomes following the introduction of durvalumab in first-line therapy for advanced BTC. In the unmatched cohort, durvalumab was associated with a substantial OS, ORR, and DCR benefit. Because cohort assignment was based on treatment era rather than randomization, we carried out propensity score matching to reduce confounding by indication; in the resulting matched cohort, durvalumab remained associated with significantly longer OS and higher ORR, with a numerically higher DCR. The attenuation of effect size after matching—from an absolute OS gain of 5.9 months (unmatched) to 3.5 months (matched)—indicates that part, though not all, of the unadjusted survival difference is attributable to baseline imbalances between treatment eras rather than to durvalumab alone.

The principal methodological strength of this study is a contemporaneously treated, chemotherapy-only comparator cohort, a feature absent from prior real-world analyses, which include only durvalumab-treated patients and cannot attribute outcomes to the immunotherapy component.^5^, ^6^, ^7^, ^8^, ^9^ However, the comparator cohort received heterogeneous first-line regimens: while all durvalumab-treated patients received a gemcitabine/platinum backbone, only 62% of the comparator cohort did. This heterogeneity constitutes an additional, chemotherapy-related source of confounding only partly addressed by including regimen as a propensity score matching covariate; residual confounding by unmeasured regimen-specific factors (e.g. dose intensity and cycle number) cannot be excluded. The comparator mOS (unmatched and matched) closely mirrored the placebo arms of TOPAZ-1 and KEYNOTE-966,^3^^,^^4^ supporting the representativeness of our comparator population despite this heterogeneity.

ORR and DCR numerically exceeded the pivotal trials in the unmatched cohort and ORR did so in the matched cohort (Supplementary Table S2, available at https://doi.org/10.1016/j.esmorw.2026.100783). We caution against interpreting this as evidence that real-world effectiveness surpasses randomized trials: more plausible explanations include favorable patient selection inherent to the nonrandomized, sequential-cohort design, treatment allocation bias, and differences in outcome assessment, since response was determined retrospectively from routine imaging rather than by independent central review, which may inflate apparent response rates. The lower DCR in our unmatched cohort likely reflects underclassification of stable disease due to nonstandardized, less frequent imaging in routine practice—illustrating that real-world response metrics are not directly comparable to trial-defined endpoints in either direction.

The TTF analysis showed a trend favoring durvalumab without reaching statistical significance (4.8 versus 3.4 months; *P* = 0.066), reflecting the known dissociation between progression endpoints and OS under ICI-based therapy. Delayed response kinetics, pseudoprogression, and prolonged postprogression benefit may each contribute to OS gains that exceed what short-term progression endpoints capture—a pattern also evident in TOPAZ-1 (PFS HR 0.75).^3^ A similar pattern was observed in the propensity-score-matched cohort (TTF 4.7 versus 3.4 months; HR 0.739, 95% CI 0.463-1.178, *P* = 0.2).

Subgroup analyses should be considered exploratory and hypothesis-generating only. With 64 patients in the durvalumab cohort, this study is clearly underpowered to formally evaluate treatment-effect heterogeneity by tumor location or sex and no formal interaction testing was carried out. The numerically greater OS difference among patients with iCCA and among male patients is directionally consistent with subgroup observations from TOPAZ-1 and KEYNOTE-966^3^^,^^4^ and with findings in other gastrointestinal malignancies (KEYNOTE-590^11^ and CheckMate 649^12^). However, given the limited sample size, wide and overlapping CIs and the observational design, these findings should not be interpreted as evidence that durvalumab is more effective in iCCA or in male patients and they do not support treatment decisions stratified by tumor subtype or sex. Prospective, adequately powered studies are needed to formally evaluate treatment-effect heterogeneity across these subgroups; the nominally significant finding in patients ≥65 years is likewise exploratory and requires independent confirmation.

Regarding safety, this analysis focused on grade ≥3 toxicities given the limitations of retrospective adverse-event documentation. The severity profile of the durvalumab cohort was broadly comparable to the chemotherapy-only cohort, with hematotoxicity the most frequent severe event in both groups, consistent with the myelosuppressive effects of gemcitabine/platinum-based chemotherapy. Grade ≥3 hepatotoxicity likely reflected a combination of chemotherapy-related liver toxicity, tumor-related hepatic impairment, and—in the durvalumab cohort—potential immune-mediated hepatitis. Immune-related adverse events of any grade were documented in 15.6% of durvalumab-treated patients, in line with TOPAZ-1 (12.7%)^3^ and published real-world series (15.6%-22.7%).^5^, ^6^, ^7^, ^8^ These data confirm that adding durvalumab to gemcitabine/platinum-based chemotherapy is feasible and well tolerated in unselected real-world patients, with a severity profile broadly consistent with TOPAZ-1^3^ and published real-world series.^5^, ^6^, ^7^, ^8^, ^9^

Several limitations merit acknowledgment. The retrospective, nonrandomized, time-period-based design precludes formal causal inference, even after propensity score matching: matching can only adjust for measured confounders, and secular improvements in supportive care, imaging technology, and second-line therapy availability over the study period may partly explain survival differences independent of durvalumab itself. ECOG performance status, an established prognostic factor, was not included in the propensity score model because it was not systematically documented across the study period and residual confounding by unmeasured differences in performance status between cohorts cannot be excluded. Despite matching, the iCCA enrichment in the durvalumab cohort (75% versus 57.4%) was not fully eliminated and may still amplify the apparent treatment effect. The comparator cohort received heterogeneous first-line regimens, as discussed above; while this was addressed by including regimen as a covariate in the propensity score model and through a sensitivity analysis restricted to patients receiving gemcitabine/platinum-based chemotherapy in both cohorts (which confirmed a significant OS benefit with durvalumab: 18.7 versus 13.9 months; *P* = 0.011; Supplementary Figure S5, available at https://doi.org/10.1016/j.esmorw.2026.100783), residual confounding by regimen-specific factors cannot be fully excluded. Objective response was assessed by retrospective review of clinically acquired imaging rather than by independent, blinded central radiological review, which may introduce assessment bias relative to trials employing prospective, standardized, blinded RECIST evaluation. Safety data are limited by retrospective ascertainment and molecular biomarkers were not systematically captured across all patients. Data were extracted from the electronic medical record into a structured database and cross-validated by a second researcher to support data quality; nonetheless, as with any retrospective real-world dataset, undetected documentation errors cannot be fully excluded. This study is reported in accordance with the ESMO Guidance for Reporting Oncology real-World evidence (GROW) recommendations and the completed ESMO-GROW checklist is provided as Supplementary Material (available at https://doi.org/10.1016/j.esmorw.2026.100783).^13^

### Conclusion

This real-world study, incorporating propensity score matching to reduce confounding by treatment era, suggests that the addition of durvalumab to gemcitabine/platinum-based chemotherapy is associated with improved OS, ORR, and DCR in advanced BTC. Given the retrospective, nonrandomized design, these findings should be regarded as supportive of, rather than exceeding, the efficacy established in TOPAZ-1 and KEYNOTE-966 and do not permit definitive causal conclusions. These data support the continued use of durvalumab-based first-line therapy in routine practice, alongside further biomarker-driven research and exploration of optimal sequencing with molecularly targeted agents.
