# Supplementary material for: Real-world survival benefit of adding durvalumab to chemotherapy in cholangiocarcinoma: a retrospective cohort analysis from a German Comprehensive Cancer Centre
Source: ESMO Real World Data Digit Oncol. 2026 Sep 21;14:100783. doi: 10.1016/j.esmorw.2026.100783 (PMC13631604; doi:10.1016/j.esmorw.2026.100783)
Supplement: Supplementary file 1 [file mmc1.pdf]

ESMO-GROW flowchart for real-world evidence studies in oncology

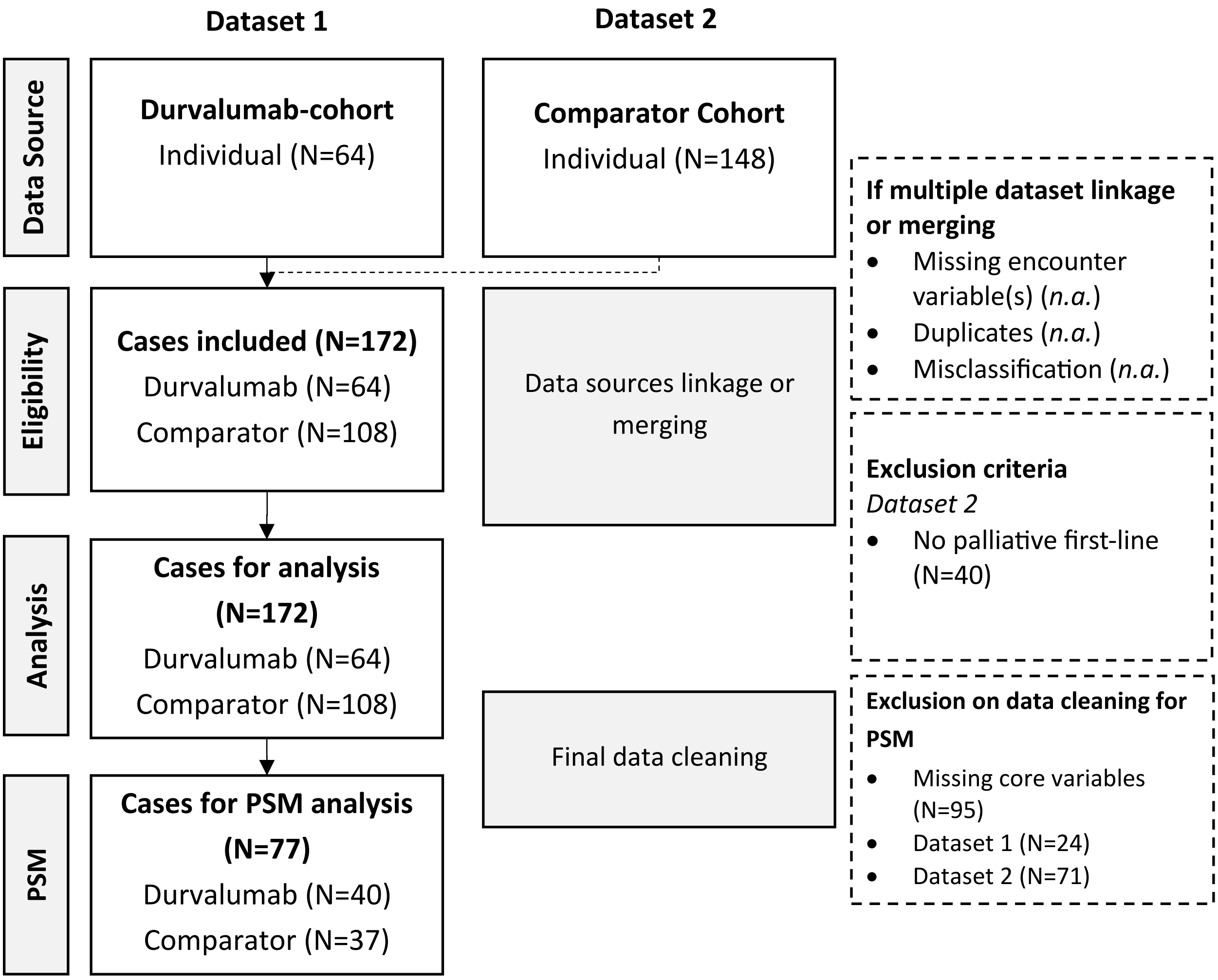

Supplemental Figure 1. ESMO-GROW flowchart for real-world evidence studies in oncology. *n.a., not applicable, PSM, propensity score matching*

A) B)

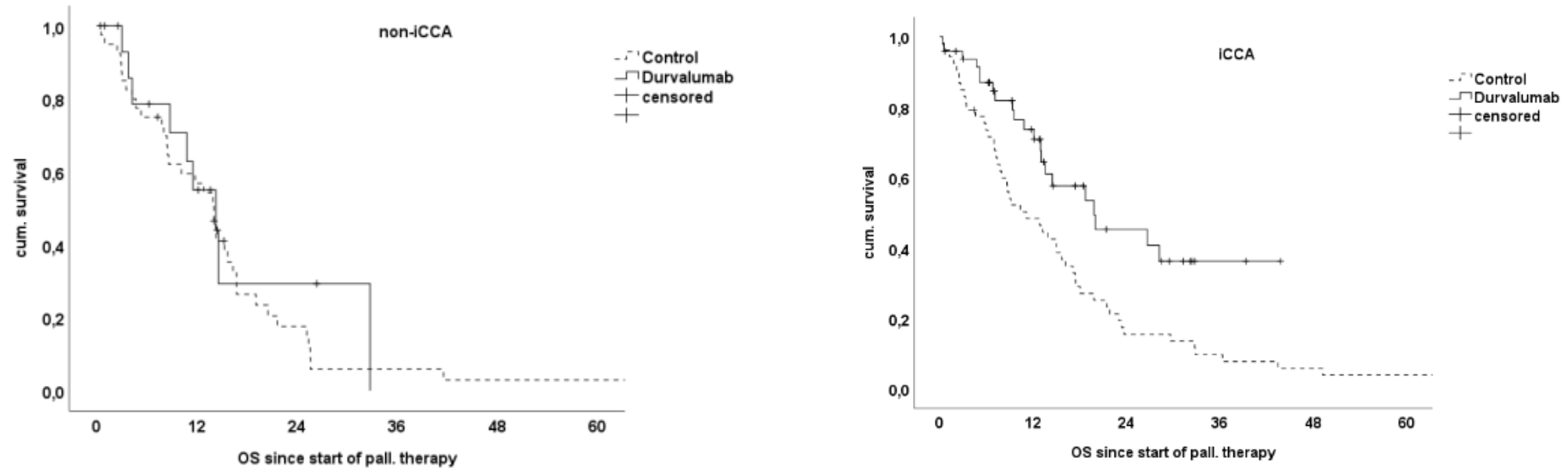

Supplemental Figure 2. Kaplan-Meier curves for OS by tumor localization. Comparison between non-iCCA (A) and iCCA (B) treated with Durvalumab or Control (dashed line). Median OS: non-iCCA in the comparator cohort 14.0 months (95% CI 11.1–17.0) vs. Durvalumab 14.2 months (95% CI 7.0–21.5); iCCA in the comparator cohort 11.1 months (95% CI 5.8–16.5 vs. Durvalumab 19.8 months (95% CI 7.1–32.5).

A)

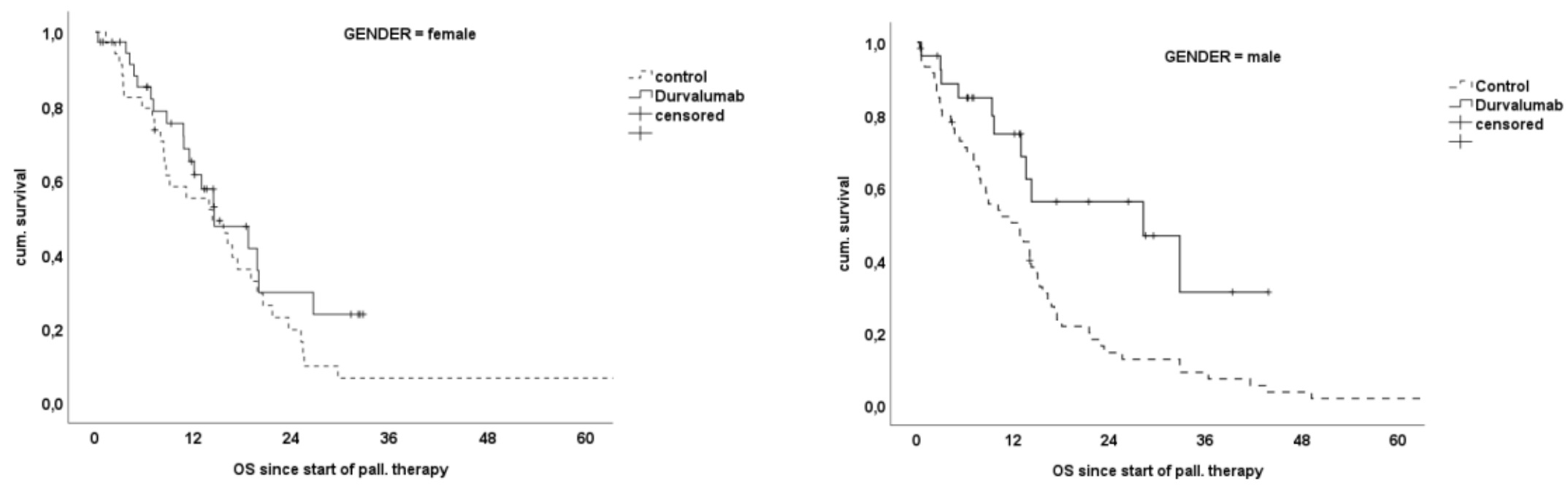

Supplemental Figure 3. Kaplan-Meier curves for OS by gender. Comparison between female (A) and male (B) patients treated with Durvalumab or Control (dashed line). Median OS: female in the comparator cohort 14.3 months (95% CI 7.5–21.1) vs. Durvalumab 14.5 months (95% CI 7.6–21.5); male in the comparator cohort 12.8 months (95% CI 7.4–18.1 vs. Durvalumab 28.2 months (95% CI 9.5–46.9).

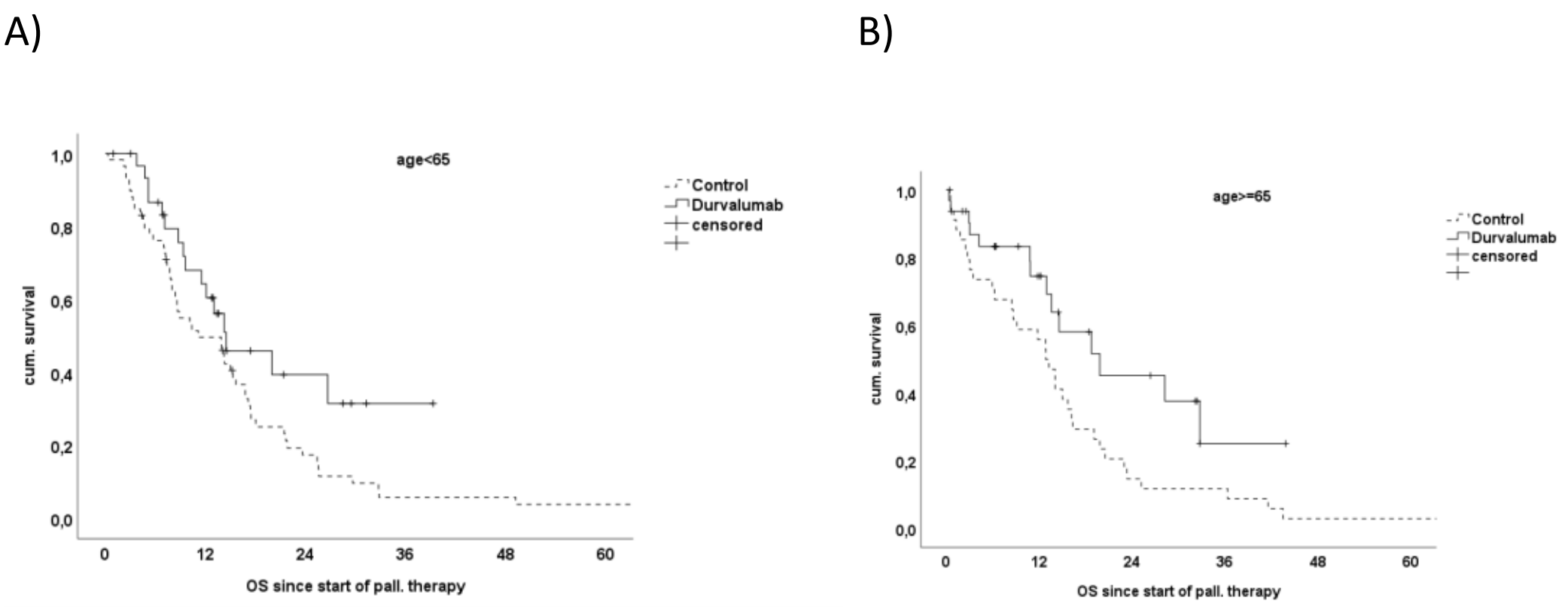

Supplemental Figure 4. Kaplan-Meier curves for OS by age. Comparison between patients <65 and ≥65 years treated with Durvalumab or Control (dashed line). Median OS: <65 in the comparator cohort 11.1 months (95% CI 5.3-17.0) vs. Durvalumab 14.5 months (95% CI 6.1–22.8); ≥65 in the comparator cohort 12.8 months (95% CI 10.2–15.4 vs. Durvalumab 19.8 months (95% CI 4.6–34.9).

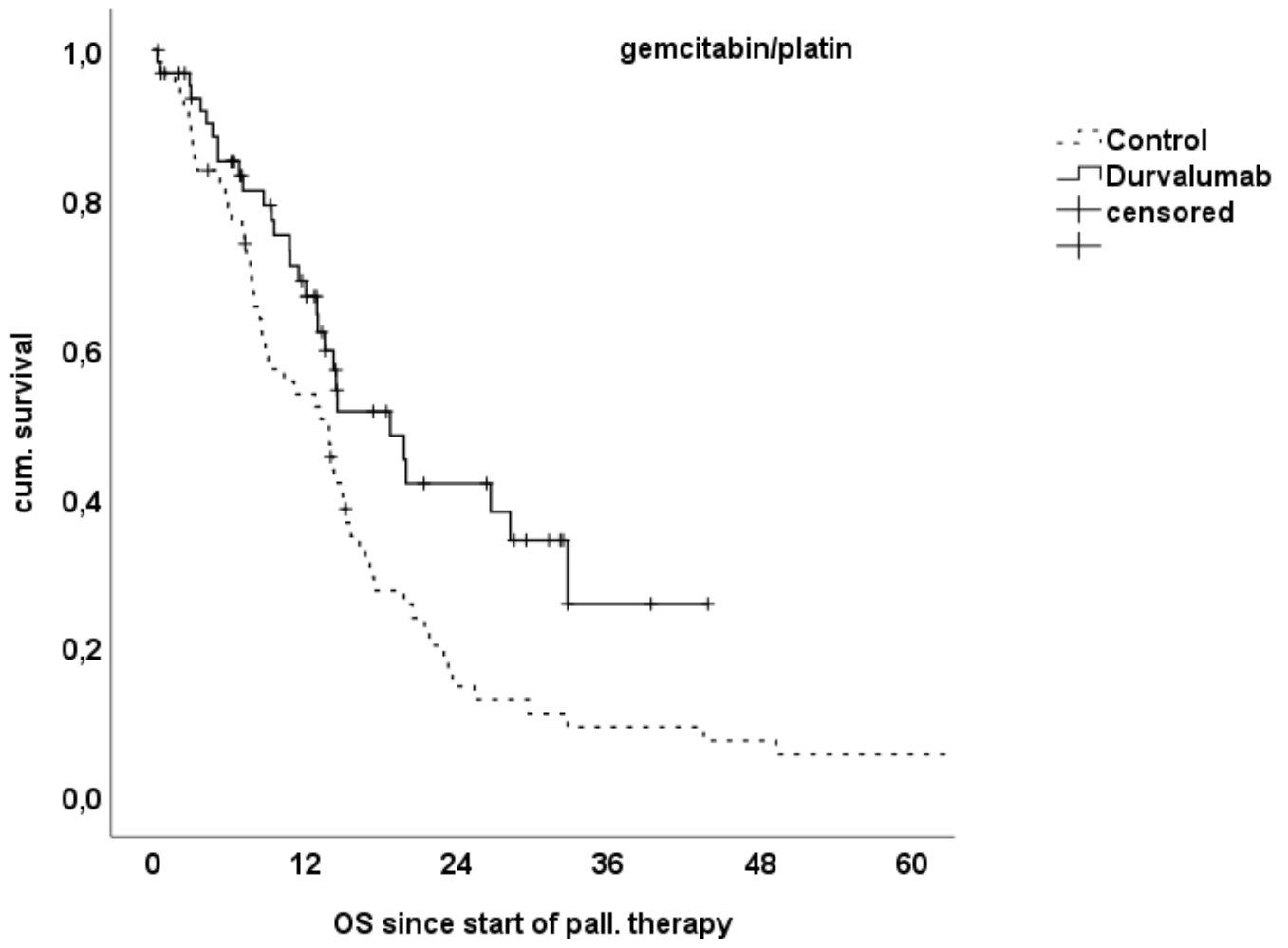

Supplemental Figure 5. Kaplan-Meier curves for OS restricted to patients treated with gemcitabine/platinum combination chemotherapy in both cohorts. Comparison between patients treated with Durvalumab or Control (dashed line). Median OS: in the comparator cohort 13.9 months (95% CI 9.8-18.0) vs. Durvalumab 18.7 months (95% CI 13.1–24.4); p=0.011.

Supplemental Table 1. Efficacy outcomes: survival and response rates after propensity score matching.

Outcome	Durvalumab (n=40)	Comparator Cohort (n=37)	p-value
**Median OS, months (95% CI)**	**14.6 (7.7–21.4)**	**11.1 (5.0–17.3)**	**0.032**
**HR OS (95% CI)**	**0.54 (0.307-0.956)**		**0.034**
**Median TTF, months (95% CI)**	**4.7 (3.6–5.9)**	**3.4 (2.1–4.7)**	0.2
**HR TTF (95% CI)**	**0.739 (0.463-1.178)**		0.203
**ORR (CR + PR)**	**45%**	**20.0%**	**0.015**
**DCR (CR + PR + SD)**	**65.0%**	**47.5%**	0.083

Abbreviations: OS, overall survival; TTF, time to treatment failure; ORR, objective response rate; DCR, disease control rate; CI, confidence interval; CR, complete response; PR, partial response; SD, stable disease

A)

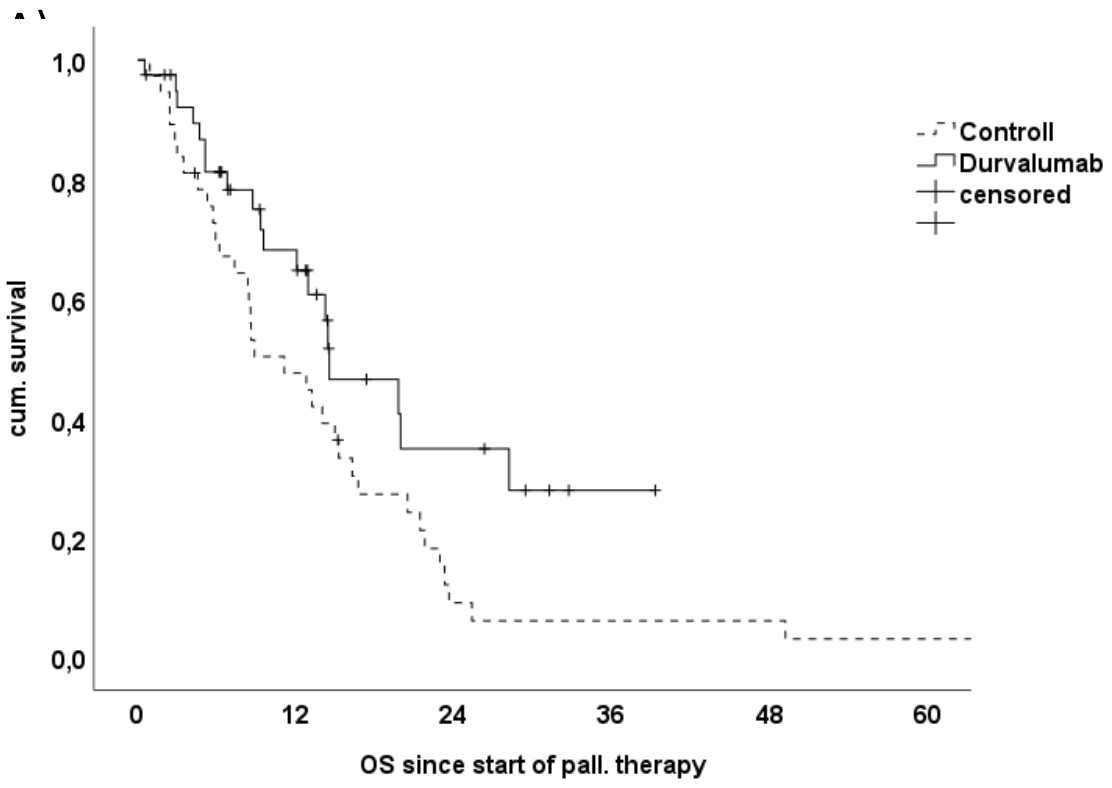

B)

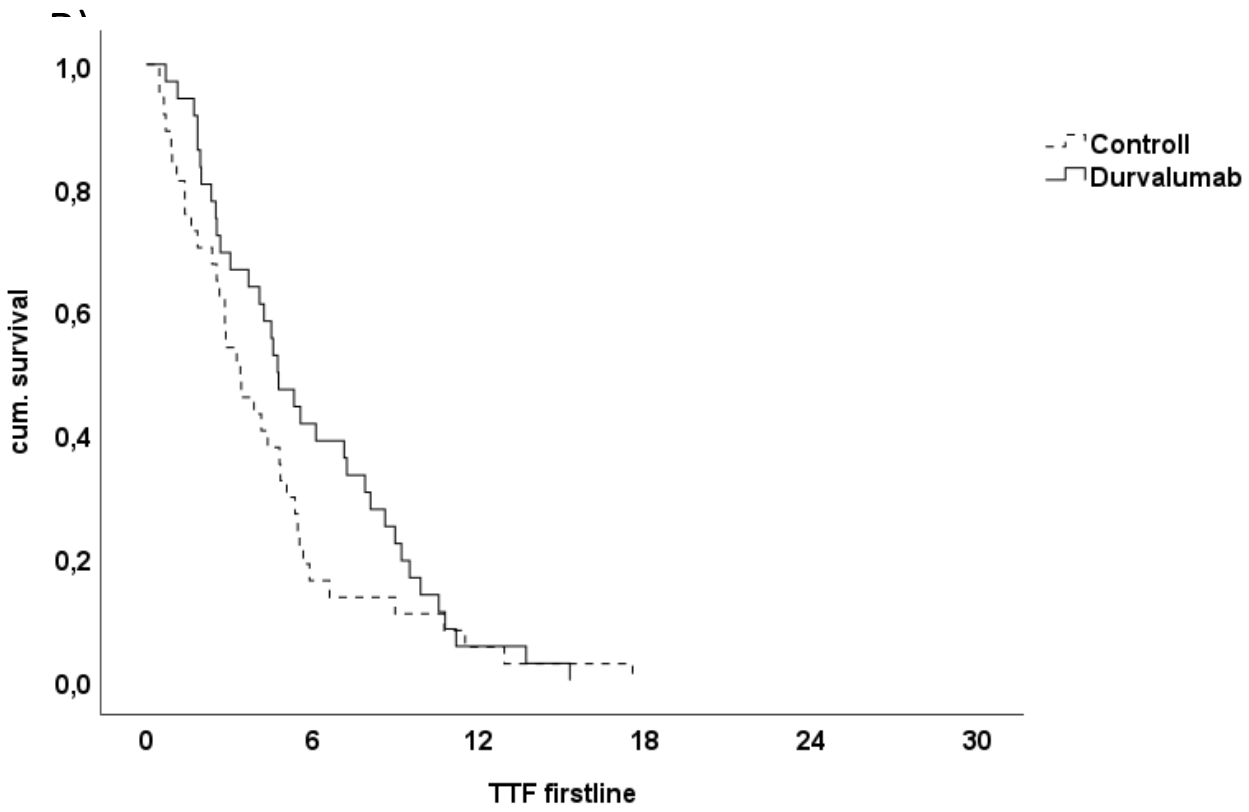

Supplemental Figure 6. Kaplan-Meier curves for overall survival (A) and time to treatment failure (TTF) (B) after propensity score matching. Comparison between the durvalumab cohort (n=40) and comparator cohort (n=37). A) Median OS: in the comparator cohort 11.1 months (95% CI 5.0-17.3) vs. Durvalumab 14.6 months (95% CI 7.7–21.4); log-rank p=0.032. B) Median TTF 3.4 months (95% CI 2.1–4.7) vs. 4.7 months (95% CI 3.6–5.9) vs.); log-rank p=0.2.

Supplemental Table 2. Cross-trial efficacy comparison: real-world cohort versus pivotal phase III trials.

Parameter	Comparator Cohort (n=108)	Durvalumab (n=64)	TOPAZ-1 Pbo (n=344)	TOPAZ-1 Durva (n=341)	KN-966 Pbo (n=536)	KN-966 Pembro (n=533)
**OS, months**	12.8	18.7	11.5	12.8	10.9	12.7
**ORR, %**	24.1	42.2	18.7	26.7	28	29
**DCR, %**	45.4	65.6	82.6	85.3	76	75

Sources: TOPAZ-1 [3]; KEYNOTE-966 [4]. Abbreviations: OS, overall survival; PFS, progression-free survival; ORR, objective response rate; DCR, disease control rate; Pbo, placebo; Durva, durvalumab; Pembro, pembrolizumab; KN-966, KEYNOTE-966.
